# A Mixed-Methods Examination of Physical Activity and Sedentary Time in Overweight and Obese South Asian Men Living in the United Kingdom

**DOI:** 10.3390/ijerph14040348

**Published:** 2017-03-27

**Authors:** Amir Emadian, Janice L. Thompson

**Affiliations:** School of Sport, Exercise and Rehabilitation Sciences, University of Birmingham, Birmingham B15 2TT, UK; j.thompson.1@bham.ac.uk

**Keywords:** physical activity, sedentary time, South Asian, Men, Type 2 Diabetes Mellitus

## Abstract

South Asian men living in the UK have higher rates of central obesity and Type 2 Diabetes Mellitus (T2DM) compared with their white British counterparts. Physical activity (PA) and sedentary time (ST) are important risk factors for the development of T2DM. The purpose of this study was to objectively measure PA, ST, and to explore the factors influencing these behaviours in this high-risk population. A mixed-methods cross-sectional research design was employed, including the quantification of PA and ST using the self-report International Physical Activity Questionnaire (IPAQ)-long form and accelerometry in overweight and obese UK South Asian men (*n* = 54), followed by semi-structured interviews in a purposive sub-sample to explore the factors influencing PA and ST (*n* = 31). Accelerometer-derived moderate-to-vigorous PA (MVPA) and ST were 298.9 ± 186.6 min/week and 551.4 ± 95.0 min/day, respectively. IPAQ-derived MVPA was significantly lower than accelerometer-derived MVPA (*p* < 0.001). IPAQ-derived ST was significantly higher than accelerometer-derived ST (*p* < 0.001). Lack of time and family commitments were identified as the main barriers to being more physically active, with group exercise identified as an important facilitator to being more active. A cultural norm of focusing on promoting education over sport participation during childhood was identified as an important factor influencing long-term PA behaviours. Work commitments and predominantly sedentary jobs were identified as the main barriers to reducing ST. Healthcare professionals and researchers need to consider the socio-cultural factors which affect PA engagement in overweight and obese South Asian men living in the UK, to ensure that advice and future interventions are tailored to address the needs of this population.

## 1. Introduction

Physical activity (PA), defined as any bodily movement produced by the skeletal muscles which expends energy [1], and sedentary time (ST), defined as any sitting or laying activities equating to less than or equal to 1.5 Metabolic Equivalent of Task (METS) [2], have both been established as individual risk factors for chronic diseases including obesity and Type 2 Diabetes Mellitus (T2DM) [1]. PA is highlighted as influential in the prevention and management of T2DM [3], with current guidelines recommending at least 150 min of moderate intensity PA and 75 min of vigorous intensity PA per week to promote general health and well-being [4,5]. In addition, epidemiological studies such as the Nurse’s Health Study have highlighted the inverse relationship between ST and T2DM risk [6], with a meta-analysis concluding that the more time adults spent being sedentary, their odds of developing metabolic syndrome increased by 73% [7].

South Asians living in the UK have substantially higher rates of central obesity and T2DM as compared to their white British counterparts, with T2DM up to six times more common in people of South Asian origin [8]. Published self-report data suggest that South Asians and other minority groups consistently undertake less PA than the majority of the population in the UK [9,10].

Self-report has been the main method for measuring PA in most population-based studies [11], and for many countries, recorded trends in changes in PA are based on a long history of questionnaire-based assessment [4]. In recent years, technological advances and reductions in costs have led to an increase in objectively measured PA using accelerometers [12]. Accelerometers are easy to wear motion sensors, which can be used to collect data to estimate the time spent in moderate-to-vigorous PA (MVPA) to determine whether individuals are meeting the minimum requirements of 150 min of MVPA per week [13].

A mixed-methods systematic review of PA in South Asians found that South Asian women had lower levels of self-reported PA when compared to both South Asian men and white European populations, and a lack of knowledge about the relative benefits of being more active [14]. Studies focusing on measuring PA and ST in South Asian men are limited; therefore there is need for these behaviours to be quantified. To our knowledge, no studies have been published to date employing a mixed-method approach to quantitatively measure PA and ST and to explore the factors that influence PA and ST behaviours in this population.

This mixed-method study will begin to address this gap in the literature and contribute to our understanding of PA and ST amongst South Asian men living in the UK by: (1) quantifying PA and ST using both self-report and objectively measured methods; and (2) using semi-structured interviews to explore factors influencing PA and ST in a sample of overweight and obese South Asian men living in the UK.

## 2. Materials and Methods

### 2.1. Study Design and Participants

A cross-sectional, mixed-methods study design was employed. Recruitment was primarily carried out through networking with community leaders and local diabetes awareness groups, which in turn helped obtain entry to mosques, temples, and community centres in the Greater London area, where it became possible to engage with members about possible participation in the study.

Participants were eligible to participate if they were of self-reported South Asian origin, were between the ages of 18 to 65 years, had not previously been diagnosed with T2DM, had a body mass index (BMI) of over 23.0 kg/m^2^ [15], and did not need assistance to walk or climb stairs. Written consent was obtained from all participants. All subjects gave their informed consent for inclusion before they participated in the study. The study was conducted in accordance with the Declaration of Helsinki, and the protocol was approved by the University of Birmingham (reference number ERN_15-0518).

A total of 63 participants were recruited between October 2015 and April 2016. The study was conducted during two separate visits scheduled one week apart. Both visits were conducted either at the participant’s home or a private location at the participant’s place of work. During the first visit, demographic and anthropometric data were gathered, and blood pressure and handgrip strength were measured. Also, participants were given an accelerometer to wear for seven consecutive days. Accelerometers were collected approximately 8–10 days later during the second visit, where self-reported PA and ST were assessed in all participants, and a semi-structured interview was conducted in a purposive sub-sample, whereby participants were chosen and interviewed until data saturation had been reached.

### 2.2. Demographic Data

Socio-demographic data including age, country of birth, ethnicity, religion, marital status, number of children, number of years living in the UK, English literacy, level of education, use of medication, and current residential postcode were collected using a self-report questionnaire. Residential postcodes were used to determine an Index of Multiple Deprivation (IMD) rank for each participant, which is an indicator of social and material deprivation in the UK [16].

### 2.3. Anthropometric Data, Blood Pressure, and Handgrip Strength

Height was measured (to the nearest mm) using the Seca 213 Stadiometer (Seca, Birmingham, UK); weight was measured (to the nearest 0.01 kg) using the Seca 899 Digital Scale (Seca). BMI was calculated by dividing weight in kg by the square of height in metres. Waist circumference was measured with a standard tape measure, with the base of the tape placed at the top of the umbilicus (to the nearest 0.5 cm). Resting blood pressure was measured using an Omron M10-It monitor (to the nearest mmHg) (Omron, Milton Keynes, UK) following 5 min of seated rest. Handgrip strength (HGS) was assessed using a Jamar hand dynamometer (to the nearest Kg Force) (Jamar, Nottingham, UK), as a measure of physical function. All measurements were taken and recorded three times, with the mean of the two closest values used in the analyses.

### 2.4. Self-Reported Physical Activity and Sedentary Time

Self-reported total moderate-to-vigorous PA and ST were assessed using the self-administered version of the International Physical Activity Questionnaire (IPAQ)-long form [17]. The IPAQ-long form is a questionnaire used to estimate the time spent in PA and ST in adults aged between 18–65 years over a seven-day period and has been validated in over 12 countries [18]. The IPAQ-long form assesses PA and ST over five different domains—‘work-related’, ‘transport’, ‘leisure time’, ‘domestic and garden’, and ‘time spent sitting’—and is therefore preferred over the IPAQ-short form for research purposes [17]. Self-reported duration (in minutes) and frequency (in days) across all five domains were then computed to produce an output for PA and ST. Based on the IPAQ protocol, the minutes per week (min/week) that were generated for PA were then multiplied by a metabolic equivalent of task (MET). METs are assigned to different forms of activity based on the level of intensity (walking = 3.0, moderate = 4.0, and vigorous = 8.0), to give a final output for PA and ST in MET–min/week.

### 2.5. Objectively Measured Physical Activity and Sedentary Time

ActiGraph GT3X accelerometers (ActiGraph, Pensacola, FL, USA) were used to objectively measure PA and ST. Data obtained from the GT3X model have been shown to be both valid and reliable across all age groups [19]. Participants were asked to wear the accelerometer on their right hip during waking hours for seven consecutive days, only removing it during time spent swimming, showering, or sleeping.

### 2.6. Semi-Structured Interviews

A purposive sub-sample of 31 participants was invited to take part in one-to-one, semi-structured interviews, which were conducted during the second visit after the participants returned their accelerometers. Participants were purposively selected to ensure maximum variation across the key demographic variables, including age, BMI, and IMD rank. An open-ended interview guide was used to facilitate the discussions and included questions relating to their understanding of what the terms PA and ST mean, the importance of PA for health and diabetes prevention, perceived barriers and facilitators to being more active, and any sociocultural factors influencing their levels of PA and ST. Pilot testing was initially carried out on three South Asian men to evaluate and revise the interview schedule prior to commencing the study; amendments were made to the schedule throughout the data collection period using an iterative process. All interviews were conducted in English, and were audio recorded and transcribed verbatim.

### 2.7. Data Reduction and Analysis

#### 2.7.1. Quantitative Data Analysis

Accelerometery data were downloaded and analysed using Actilife 6 software (v6.8.2, Actigraph, LLC, Pensacola, FL, USA). An epoch of 60 s was chosen for analysis [18]; a minimum of 600 min wear time was used as the cut-off point for valid data, with a minimum of four days of valid data required to be included in analyses [19,20]. Non-wear periods were defined as greater than 60 consecutive minutes with zero activity counts. The Freedson et al. [21] cut-off points was used to determine the time spent in various levels of PA (0–99 counts/min = sedentary, 100–1951 counts/min = light intensity activity, 1952–5724 counts/min = moderate intensity activity, 5725–9498 counts/min = vigorous intensity activity, and >9499 = very vigorous intensity activity). These cut-off points have previously been used in South Asian populations and therefore, were deemed appropriate for this study and allowed for comparability with the published data [22,23].

As the IPAQ-long form provides an output in MET–min/week, accelerometer data were also converted to MET–min/week (4 × min of moderate intensity activity + 8 × min vigorous intensity activity) for the purpose of comparing accelerometer-derived to IPAQ-derived MVPA.

As data were not normally distributed, a two-step approach was used to normalise the data before statistical analyses were conducted [24], with an initial step of transforming the variables into percentile ranks, followed by a second step of operating an inverse normal transformation to the results from the first step. Descriptive analyses (mean, standard deviation, range, percentage) were conducted for all variables. Independent *t*-tests were performed to compare demographic variables between the full sample and the purposive sub-sample that participated in the interviews. Pearson’s correlations were used to examine the relationships between accelerometer-derived MVPA and ST, and IPAQ-derived MVPA and ST. Bland-Altman plots were used to explore the differences between accelerometer- and IPAQ-derived MVPA and ST, with dependent *t*-tests used to examine if differences between self-reported and objective measures significantly differed from zero. The effects of the predictor variables of MVPA and ST were explored using multiple linear regression using the enter method. All statistical analyses were conducted using IBM SPSS statistical analysis software package (Version 22.0; IBM, Armonk, NY, USA).

#### 2.7.2. Qualitative Data Analysis

Direct content analysis was used to analyse qualitative interviews. This particular approach is suitable for qualitative data analysis when there is pre-existing research on the phenomenon being investigated [25]. In this instance, there is published research on the motivations and facilitators of PA in overweight and obese adults, in addition to their being existing data on perspectives of the use of PA for health promotion in South Asian men [26,27]. The existing research not only contributed to choosing the method of analysis but also steered the process for constructing the interview guide. During the course of developing the interview guide, key themes and concepts emerged, which were then used to build the main coding categories for analysis. All transcripts were coded using the predetermined categories, while a second independent researcher coded a selection of transcripts to ensure agreement in coding. Data that did not fit within the initial coding scheme were assigned as a new code. Once a final coding frame was established, data were re-examined and coded based on the final coding categories.

## 3. Results

### 3.1. Quantitative Results

Fifty-four of the 63 participants (85.71%) recruited into the study met the minimum of four days of valid accelerometery data and were included in the analysis, with 31 participants agreeing to take part in a semi-structured interview. Demographic, anthropometric, blood pressure, and HGS data are reported in Table 1, with no differences found in these variables between the 63 participants who were recruited, the 54 participants with valid data, and the 31 participants who participated in the interview. As reported in Table 2, participants represented a range of different ethnic and religious backgrounds, with the majority being Indian (71.4%) and Hindu (50.8%).

As reported in Table 3, accelerometry-derived data indicated that the participants engaged in 298.9 ± 186.6 min/week of MVPA, with 33 participants (61.1%) recording at least 150 min of MVPA/week. However, only 13 (24.1%) met the recommendation of 150 min or more of MVPA in 10 or more minute bouts. Accelerometer-derived ST indicated that participants spent 551.4 ± 95.0 min/week being sedentary, with a mean of 65.3 ± 8.6% of time spent being sedentary.

One-way ANOVA analysis reported no significant differences in accelerometer-derived MVPA between the demographic variables of levels of education, ethnicity, or country of birth (*p* = 0.171, *p* = 0.761, and *p* = 0.666, respectively), or ST and the same variables (*p* = 0.184, *p* = 0.536, and *p* = 0.359, respectively).

As shown in Table 4, IPAQ-derived MVPA was significantly lower than accelerometer-derived MVPA. In contrast, IPAQ-derived ST was significantly higher than accelerometer-derived ST. Pearson’s correlation indicated a moderate significant positive relationship between IPAQ-derived MVPA and accelerometer-derived MVPA (r = 0.414, *p* = 0.002), and a moderate positive correlation between IPAQ-derived ST and accelerometer-derived ST (r = 0.446, *p* = 0.001). Furthermore, tests comparing Total MVPA, IPAQ MVPA, Total Sedentary, and IPAQ sedentary time between the 54 participants included in analysis and the 31 participants in the purposive sub-sample (herewith referred to as the sub-sample) included in qualitative analyses showed no significant differences between the variables.

**Figure 1:**
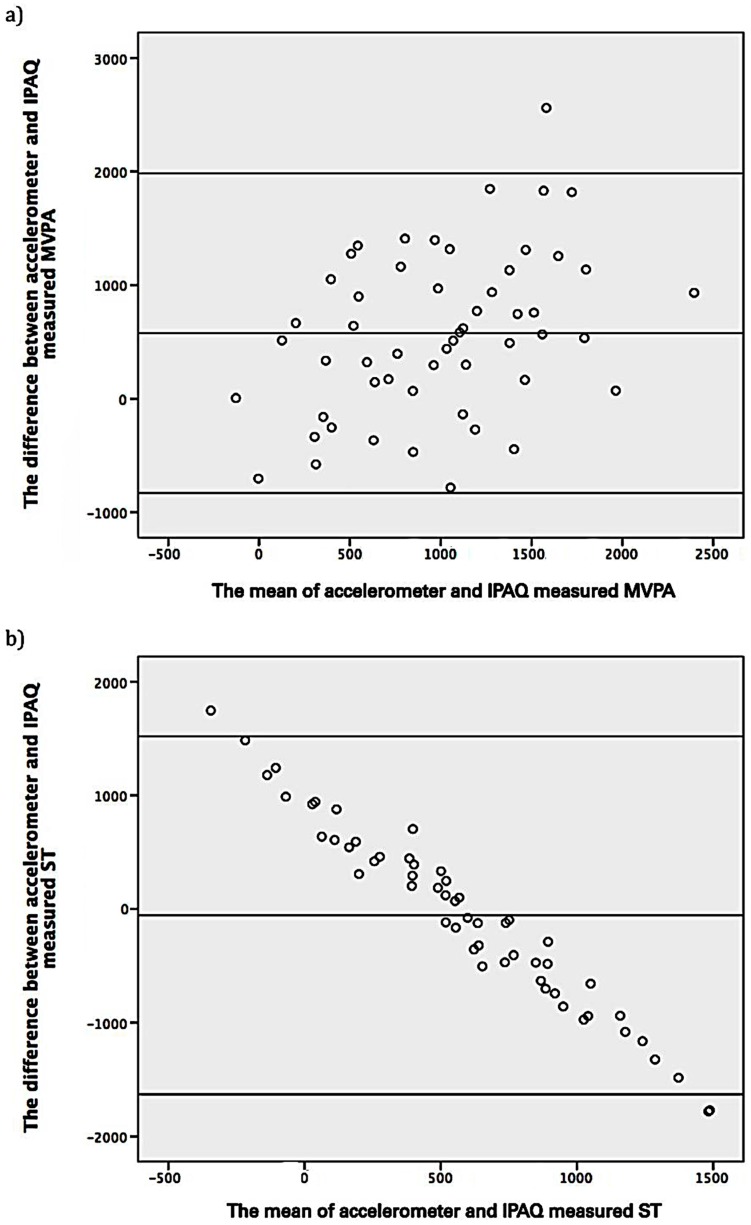
(**a**) The difference between accelerometer and IPAQ measured MVPA (*y*-axis) plotted against the mean difference of accelerometer and IPAQ measured MVPA (*x*-axis) with 95% limits of agreement. Overall mean difference was 578.2 MET–min/week and the limits of agreement were −828.7 to 1985.1; (**b**) The difference between accelerometer and IPAQ measured ST (*y*-axis) plotted against the mean difference of accelerometer and IPAQ measured ST (*x*-axis) with 95% limits of agreement. Overall mean difference was −56.2 MET–min/day and the limits of agreement were −1631.2 to 1518.9. Data shown are normalised using the two-step approach.

Bland–Altman plots showing the difference between IPAQ and accelerometer-derived MVPA and ST are shown in Figure 1. The mean difference between IPAQ-derived and accelerometer-derived MVPA was 578.2 MET–min/week (*p* < 0.001) (values are normalised using the two-step approach) and the 95% limits of agreement were relatively broad (−828.7 to 1985.1). For ST, the mean difference was −56.2 min/day (*p* = 0.610) (values are normalised using the two-step approach) and the 95 % limits of agreement were also relatively broad (−1631.2 to 1518.9).

Multiple linear regression models using socio-demographic variables as predictors for accelerometer-derived MVPA resulted in no significant models, however, for accelerometer-derived ST, the IMD rank explained 4.6% of the variance in ST (F (1, 48) = 4.190, *p* = 0.046), suggesting that participants living in the least deprived areas have higher levels of ST than those living is more deprived areas.

### 3.2. Qualitative Results

**Figure 2:**
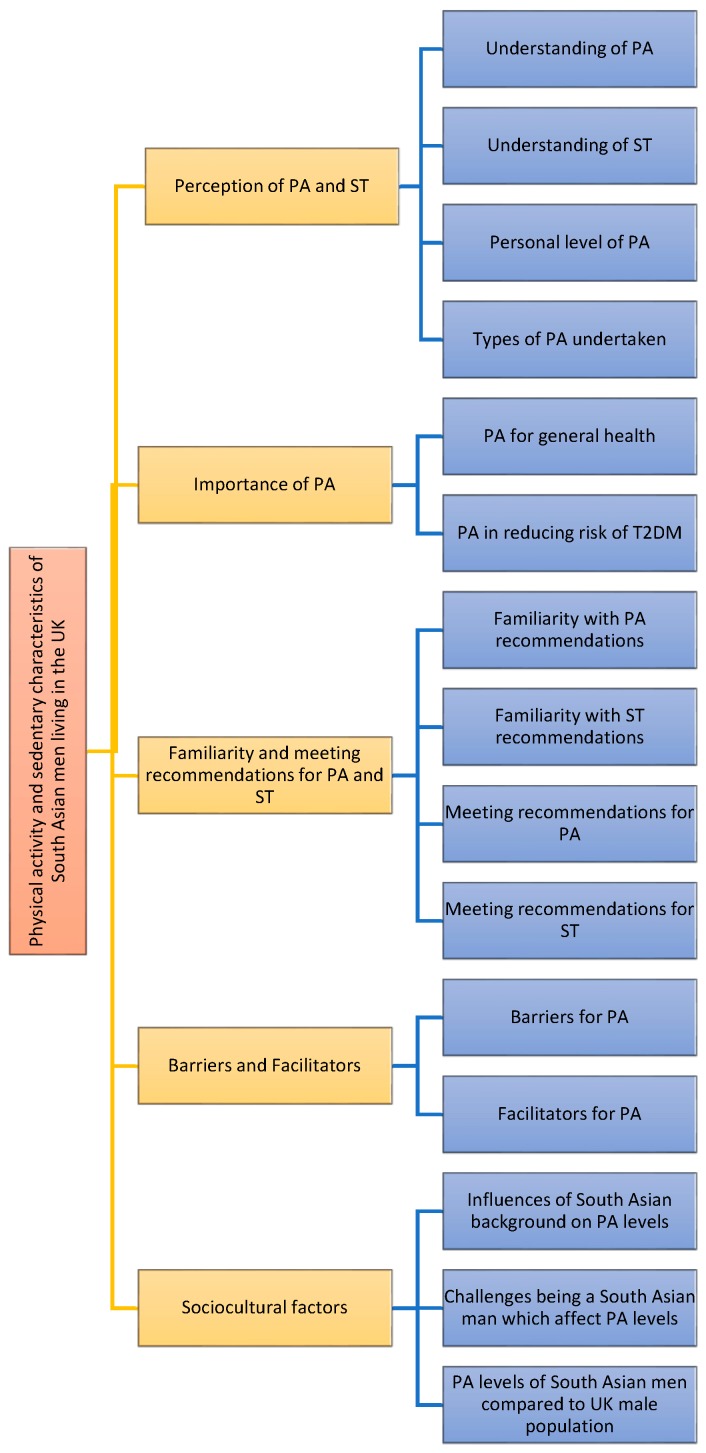
Diagram showing the final coding matrix identifying the key factors identified as influencing physical activity and sedentary time in the 31 participants who participated in the semi-structured qualitative interviews. T2DM: Type 2 Diabetes Mellitus.

Figure 2 illustrates the final coding matrix of the key factors reported to affect PA and ST in the qualitative interviews.

The following sections describe the findings within each code, supported by representative quotes.

#### 3.2.1. Understanding of Physical Activity

Participants conceptualised PA in two different ways, with half of the men considering PA to be any form of movement:

You just keeping moving. I would say walking, cycling, any household chores, any games or extracurricular activities. I guess even walking, standing up and sitting down is also considered physical activity.

47 years, Indian, Hindu, living in the UK for 17 years

Whereas the other half of interviewees considered PA to be more than just general movement, often referring to more intense forms of activity:

Physical activity for me, I think is more than just walking or sitting down would be to do some extra physical activity like exercise or… something out of your comfort zone.

30 years, Indian, Sikh, living in the UK for 5 years

#### 3.2.2. Understanding of Sedentary Time

Over half of the participants (17/31) were not familiar with the term ‘sedentary time.’ Those who were aware of this term demonstrated a comprehensive understanding:

It’s just like sitting on a chair reading or watching tele [television] or something like that. Sedentary…that is just stationary. Your body is stationary. Maybe [a] little hand movement may be there, otherwise that is, that also could be considered as sedentary.

63 years, Indian, Hindu, living in the UK for 19 years

#### 3.2.3. Personal Level of Physical Activity

Over half the men (17/31) described their own level of PA as inactive. The majority of men (21/31) stated that their work commitments were the main reason for the difference in weekday to weekend activity levels. As one participant explained:

When I’m at work then I’ll say I’m not very active that’s just the way that my lifestyle is at work, because most of the time I am behind my desk.

25 years, Indian, Sikh, living in the UK for 25 years

#### 3.2.4. Types of Physical Activity Undertaken

Overall walking was reported as the most common form of PA:

[I] walk, very fast, as fast as I can, right, so that, that’s what I try to do. Me and my wife both, both are very similar thinking; we try to do that.

42 years, Indian, Hindu, living in the UK for 11 years

Other activities mentioned included running, swimming, cycling, badminton, and football. Badminton in particular was revealed to be one of the more favoured activities to play over the weekends, as it was mentioned by 35.5% of those interviewed:

And over the weekend I do make a point to go for badminton for like an hour or an hour and a half, or sometimes two hours. So for example, this Sunday I played for two hours.

37 years, Indian, Hindu, living in the UK for 5 years

#### 3.2.5. Physical Activity for General Health

All of the men considered PA to be important for general health, in particular in relation to promoting weight loss, with some also referring to the perceived benefits for cardiovascular health:

Well, because it... physical activity helps me to bring down my fat. That’s what I’m trying to do actively and then it helps in pumping the heart, which in turn helps in the blood circulation which in turn helps reducing the fat deposits and cholesterol and everything.

45 years, Indian, Hindu, living in the UK for 11 years

The benefits of PA in promoting mental health was also highlighted by some:

I think (it’s) very important. Not even [just for] your physical health but your mental health leaves a lot of stress, but with regards to physical health, yes.

27 years, Indian, Sikh, living in the UK for 27 years

Another theme which emerged was the perceived increased importance of PA with age:

Because as you go along with your age, you get old, you’ve got more chances to get any kind of disease. So you need to be having more physical activities, to keep fit yourself, so you can stay away from this type of disease…

49 years, Indian, Sikh, living in the UK for 30 years

#### 3.2.6. Physical Activity in Reducing Risk of Type 2 Diabetes Mellitus

Apart from three participants who were unsure, the remainder of those interviewed were confident that PA helped reduce the risk of T2DM, with most attributing the benefits to the role of PA in helping to reduce body weight:

Yes, definitely. Well, first of all it would help me lose my weight, and that I think should have an impact on everything on my health overall and including diabetes.

47 years, Indian, Hindu, living in the UK for 17 years

Others made reference to how they thought PA would affect blood glucose levels:

Because you are basically, burning all the calories, so you’re not, accumulating any, cholesterol or fat in the body, so by doing more (physical activity), you know, burning the energy out, you need less insulin to digest, yeah, otherwise, you need more insulin in the body to get rid of the sugar to compensate.

47 years, Indian, Hindu, living in the UK for 15 years

#### 3.2.7. Familiarity with Recommendations

Only 5 of the 31 (16.1%) participants were familiar with the PA recommendations of achieving at least 150 min of MVPA or 75 min of vigorous activity per week in bouts of 10 min or more. None of the participants were familiar with the current ST recommendations of minimising ST.

#### 3.2.8. Meeting Recommendations

After describing the PA and ST recommendations to the participants, 13 (42.0%) thought they were meeting the recommendation for PA, with only 6 (19.4%) believing they were meeting the recommendation for ST.

#### 3.2.9. Barriers to Physical Activity

A lack of time was the main theme identified as a barrier to being more active. This was subdivided into a lack of time due to work or family commitments:

Barriers to me, yeah, it’s my work. I have to be in my office and have to be available because I work with, ah, overseas customers, so they expect me to be there, and then I just can’t, you know, make time to get out.

51 years, Indian, Hindu, living in the UK for 23 years

Yeah, just now is the timings you know, dropping the kids you know, at various locations cause my daughter started school now, recently in September and my son’s going nursery so my wife’s got to drop my daughter and I have to drop my son. So that’s taking too much time and by the time I finish there’s no time to do exercise.

30 years, Indian, Sikh, living in the UK for 5 years

Some participants also considered the weather to be a barrier, stating that the poor weather in the UK made it more difficult for them to live more active lives:

Also because of the climate I can’t walk as often as I would like to here.

41 years, Indian, Hindu, living in the UK for 10 years

#### 3.2.10. Facilitators of Physical Activity

Group exercise was the main theme that emerged as a facilitator to being more active, with 11 participants citing the motivating influence of exercising with others:

Also in a group level, I find that very, very helpful. Obviously when there’s other people around, that helps, and it has a kind of a knock-on positive effect as well.

58 years, Indian, Hindu, living in the UK for 54 years

However five men considered personal responsibility to be the most important factor in being more active:

But I think it is very, very personal driven. As an individual, I know what good it is, and I know what I should be doing.

37 years, Indian, Hindu, living in the UK for 5 years

#### 3.2.11. Influence of South Asian Background

Twenty-four out of the 31 participants (77.4%) believed that their South Asian background has an influence on their PA levels. A prioritisation of academia rather than sports during childhood was the focal point of discussion. As one man explained:

Because it is not embedded from the childhood that physical activity is, kind of, a must. We are being programmed and attuned to be academic, more academic, and then doing either valid business or in the career path or kind of thing, so it’s more about academic, not much emphasis about physical activity. You know, when I grew up, sports was seen as a waste of time—I can’t earn money [engaging in sports]. So it’s all about keeping yourself, you know, ahead, for survival.

(47 years, Indian, Hindu, living in the UK for 15 years)

Nonetheless, many believed that circumstances have changed since they were growing up, often drawing comparisons with their own children:

But of course, it’s more of the socio-economic structure at that time, we're talking about the ‘70s and ‘80s. So then it was completely different [In India]. But whereas now, when I see the [UK] population in comparison, I think people are equally aware, of the importance [of physical activity]…whereas I take my kid for a particular thing or for different lessons, he goes for cricket, for badminton, swimming, football, so I encourage him to go for many more activities.

47 years, Indian, Hindu, living in the UK for 16 years

The family-orientated nature of South Asian communities was another theme identified as influencing PA levels, with participants reporting that they believed that South Asian men are more dependent on their families compared to other men in the UK:

Mostly family responsibilities, like, here, people here [in the UK] are very independent, whereas in South Asia they’re more family oriented. Like, they live with their parents, so they live with their families, so they will have more get-togethers, gatherings, so... and they tend to, go in the cars every time so they don’t tend to walk a lot, I mean, not do any kind of physical activities.

40 years, Indian, Hindu, living in the UK for 2 years

#### 3.2.12. Physical Activity Levels of South Asian Men Compared to UK Men

The majority (29/31) considered South Asian men living in the UK to be less physically active than the general UK male population, with only two participants believing the contrary. Two participants felt that PA levels were not necessarily different due to ethnicity, but more dependent upon the individual’s work environment, as the following quote illustrates:

They’re [Asian men] different, again, it all comes down to their, sort of professional lifestyle a lot of Asian men are you know, if in finance or business you’re sitting down a lot, but then a lot of them are in construction or property that do work very vigorously you know, physical work and, no nothing like that, it comes out all down to your routine and your job.

30 years, Indian, Sikh, living in the UK for 5 years

## 4. Discussion

Using a mixed-methods design, this study quantitatively and qualitatively examined the PA and ST of overweight and obese South Asian men living in the UK. This study provides an important and unique contribution to the literature, and to our knowledge it is the first study to examine the comparability of accelerometer-derived PA and ST to the IPAQ-long form in this population, in addition to being the first to objectively measure ST and to conduct qualitative interviews exploring factors affecting the PA and ST levels in this population.

The mean MVPA and ST for our sample were 298.94 min/week and 551.40 min/day, respectively. Celis-Morales et al. [22], reported lower MVPA levels in South Asian men living in the UK (181.3 min/week), as did Yates et al. in their mixed sample of UK South Asians (126 min/week) [28]. Comparisons with data from the Health Survey for England (HSE) [29] show that mean MVPA in our sample was higher than the 217 min/week reported for the general male adult population, with the mean ST for our sample being considerably higher (551.4 min/day vs. 309 min/day) (Health and Social Care Information Centre 2015) [30]. Furthermore, mean PA and ST levels in our sample were more comparable to data from a sample of South Asian women living in the UK (242.6 min/week of MVPA and 530.2 min/day of ST) [23].

Although there was a moderate significant positive correlation between IPAQ and accelerometer-derived MVPA and ST, the IPAQ significantly underestimated MVPA and overestimated ST. Furthermore as demonstrated by the Bland–Altman plots, the 95% limits of agreement were relatively broad, indicating that the two methods were not likely to be measuring MVPA and ST similarly, therefore the IPAQ-long form may not be suitable for use in this population. Yates et al. measured MVPA in South Asian men and women from a similar age range to our sample, reporting a similar positive correlation between accelerometer and IPAQ-derived MVPA. However, in their sample, self-reported MVPA levels were significantly higher than objectively measured MVPA, as assessed using the IPAQ-short form [28]. Due to having fewer input options for the specific activity domains, the short-form can potentially overestimate PA levels [31]. Results from multiple linear regression analyses suggest that men from least deprived areas had higher levels of ST. This may be in part because people from less deprived areas are more likely to have office-based jobs and hence would spend more time being sedentary [29,32].

The qualitative data in the present study suggest that all of the men who participated in interviews were aware of the importance of PA for general health, and had a good understanding of the role of PA in reducing the risk of T2DM. Only 16.1% of the men interviewed were familiar with the UK guidelines for PA; this value is higher than the 6% of the general male population in the UK who are familiar with current PA guidelines [30].

A lack of time due to work and family commitments was reported as the main barriers to being more active. This is a commonly reported barrier to PA in adults and is not unique to South Asian adults [33]. One important cultural aspect identified in this study is a relatively high level of family commitments, with an emphasis on the expectation that South Asian men prioritise time away from work to spend it with their families as opposed to engaging in leisure time PA. This finding has also been identified in previously published studies of South Asian men [34,35,36]. Participants also identified the weather as a barrier to being more active, with poor weather in the UK thought to limit their outdoor activity, which is consistent with existing research on South Asian adults living in colder climates [37], in addition to being a common barrier reported by adults across various ethnic minority groups [38,39].

In the present study, participating in exercise with others was reported to be the primary facilitator to being more active. This theme is supported by previous research in South Asian adults [26,27,36]; these studies reported preferences for South Asian adults to socialise while taking part in PA. It is important to note that engaging in exercise with others is a common facilitator for PA in many populations, and is not exclusive to South Asians [33,40].

A unique culture-related finding from our data was the concept that the men felt that their upbringing within a South Asian family placed a greater focus on education over exercise, ultimately influencing their PA levels as adults. Many participants referred to the importance of education in South Asian families and countries as a means to achieve financial stability. In particular, those who grew up in a lower socioeconomic environment in India commented that education was considered the main route to improving quality of life. Hence the cultural lack of focus on PA experienced as children may have impacted the formation of habits and long-term PA behaviours. Some also made cross-cultural comparisons to the higher importance placed on PA in the UK overall, compared to less importance being placed on PA in South Asian countries. This finding is consistent with published research emphasising the development of early childhood habits in relation to being physically active, which in turn will affect PA behaviours in adulthood [41,42,43]. Some participants in this study suggested that this cultural attitude to PA has begun to change within South Asians who are born and raised in the UK. Their views are supported by research indicating that second-generation South Asians may have a more favourable attitude towards PA compared to first-generation South Asians living in the UK [44]. Participants also revealed how they have provided support for their children to engage in more PA, using their own childhood as a reference point when describing the difference in the amount of extracurricular PA they encourage their own children to take part in, compared to when the participants themselves were children.

Recent evidence suggests that new physical activity recommendations specific to South Asians may be needed to account for this population’s increased risk of developing T2DM and other metabolic diseases. Iliodromiti and colleagues [45] investigated the level of MVPA that South Asian adults required to match the same cardio-metabolic risk profile observed in white Europeans of similar age and BMI undertaking the recommended 150 min of MVPA/week. The results of this study indicated that South Asian adults need to be undertaking at least 232 min/week of MVPA to achieve the same cardio-metabolic benefits that white European adults achieve by engaging in 150 min/week. In the present study, the mean min/week of MVPA of South Asian men exceeded the 232 min/week threshold suggested by Iliodromiti et al. [45], with 33 participants (61.1%) exceeding this threshold. Although the findings of Iliodromiti et al. need to be replicated in a larger sample, they draw attention to the potential need for physical activity guidelines that are tailored for groups at increased risk for metabolic diseases such as T2DM and cardiovascular disease.

This study was not without limitations. The sample size was relatively small, and therefore is likely not representative of South Asian men living in the UK, which limits the generalizability of our results. In addition, 81.5% of our participants were university educated, which may also limit the generalizability of our findings to the wider population. However it is important to note that recent research suggest that Indians of Sikh and Hindu faith are more likely to achieve higher levels of education than other South Asian groups, and as such our findings provide insights into this sub-group within the economically and culturally diverse classification of ‘South Asians’ in the UK [46].

One strength of our study was objectively measuring PA and ST to allow for comparisons with self-report data. Another strength of this study was the recruitment of individuals who have traditionally been defined as ‘hard-to-reach’, along with the inclusion of South Asian men across a range of ages, faith groups, and countries of origin. Upon initiating this research, we believed that an additional strength of this study was incorporating the use of the long-form version of the IPAQ instead of the short-form version used in previous research [23,28]. Based on previous research, it was expected that the long-form version would provide a more accurate estimate of MVPA than the short-form, but our findings suggest this was not the case. As such, it appears that the utility of both the short- and long-form versions of the IPAQ may be limited in this population.

As our results indicate that exercising with others is a main facilitator to engaging in PA in this sample, future intervention programmes encouraging group exercise should be promoted as a strategy for increasing PA levels. Based on the participants’ responses, team sports such as group badminton, football, and cricket appeared to be favourable to the men, and as such could be implemented in community and faith centres to increase participation and acceptance. Furthermore, given the high levels of objectively measured ST and the lack of awareness about recommendations to reduce ST, future intervention should focus on awareness campaigns in community centres and places of worship to promote the importance of ST reduction.

## 5. Conclusions

Our data indicate that this sample of South Asian men are more physically active than has been previously reported in studies using objectively measured methods. Nonetheless, most are not meeting the recommendation of 150 min of MVPA per week in 10 or more minute bouts. However, more data are needed to determine whether this holds true in a larger, more representative sample of South Asian men, and to compare their MVPA levels to those of the general UK population. The results suggest that the IPAQ-long form may not accurately measure MVPA and ST in South Asian men living in the UK. This study revealed additional information about the unique socio-cultural factors which affect PA engagement in overweight and obese South Asian men. It is crucial that healthcare professionals and researchers understand these behaviours in order to tailor advice and future intervention to address the needs of this population.

## Figures and Tables

**Table 1:** Anthropometric characteristics, resting blood pressure, handgrip strength, and index of multiple deprivation data for 63 South Asian men participating in the study.

	Full Study Sample (A) (*N* = 63)	Sample with Valid Data Included in Analyses (B) (*n* = 54)	Sub-Sample Participating in Qualitative Interview (C) (*n* = 31)	*p*-Value Differences between A and B	*p*-Value Differences between A and C	*p*-Value Differences between B and C
Age (years)	44.8 ± 9.9	45.0 ± 9.79	43.9 ± 10.4	0.449	0.728	0.569
BMI (kg/m^2^)	28.1 ± 4.2	27.5 ± 3.1	27.8 ± 3.7	0.816	0.618	0.619
Weight (kg)	82.0 ± 13.0	80.1 ± 10.7	80.8 ± 11.9	0.910	0.354	0.674
Height (cm)	170.8 ± 6.1	170.7 ± 6.1	170.3 ± 6.6	0.715	0.605	0.960
Waist Circumference (cm)	99.7 ± 10.4	98.2 ± 8.6	99.2 ± 9.4	0.242	0.758	0.810
Systolic Blood Pressure (mmHg)	128.2 ± 15.2	128.2 ± 15.9	128.4 ± 16.1	0.687	0.581	0.777
Diastolic Blood Pressure (mmHg)	84.8 ± 9.4	84.7 ± 9.8	84.4 ± 10.4	0.502	0.524	0.786
HGS (kg)	29.9 ± 6.6	29.7 ± 6.9	30.4 ± 7.3	0.890	0.504	0.766
IMD rank	16,661.5 ± 9470.8	16,907.3 ± 9642.3	19,818.5 ± 8281.2	0.102	0.147	0.298

BMI: Body Mass Index; HGS: Handgrip Strength; IMD: Index of Multiple Deprivation.

**Table 2:** Demographic characteristics of the 54 South Asian men with valid accelerometry data.

Characteristic	Number (%)
**Country of birth**	
UK	9 (16.7)
India	39 (72.2)
Pakistan	1 (1.9)
Bangladesh	5 (9.3)
**Ethnicity**	
Indian	48 (88.9)
Pakistani	1 (1.9)
Bangladeshi	5 (9.3)
**Faith**	
Hindu	26 (48.1)
Sikh	17 (31.5)
Muslim	7 (13.0)
Hare Krishna	2 (3.7)
Buddhist	2 (3.7)
**Education**	
University/Higher Education	44 (81.5)
College	6 (11.1)
Secondary School	3 (5.6)
Primary School	1 (1.9)
**Self-Reported English Literacy**	
Excellent	31 (57.4)
Good	16 (29.6)
Fair	6 (11.1)
Poor	1 (1.9)
**Self-Reported Health**	
Excellent	8 (14.8)
Good	31 (57.4)
Fair	14 (25.9)
Poor	1 (1.9)
**Taking Prescribed Medication**	
Yes	23 (42.6)
No	31 (57.4)
**Taking Blood Pressure Medication**	
Yes	14 (25.9)
No	40 (74.1)

**Table 3:** Summary of accelerometery measured physical activity in 54 South Asian men with valid accelerometery data.

Variables	Mean ± SD
Moderate Intensity PA (min/week)	284.6 ±183.9
Vigorous Intensity PA (min/week)	14.2 ± 26.1
Very Vigorous Intensity PA (min/week)	0.1 ± 0.5
Total MVPA (min/week)	298.9 ± 186.6
Sedentary Time (min/week)	551.4 ± 95.0
% Time Spent Sedentary	65.3 ± 8.6
	***n* (%)**
Participants achieving 150 min of MVPA	33 (61.1)
Participants achieving 150 min of MVPA in 10 or more minute bouts	13 (24.1)

SD: Standard Deviation; PA: Physical Activity; MVPA: Moderate-to-Vigorous Physical Activity.

**Table 4:** Comparison of International Physical Activity Questionnaire (IPAQ) and accelerometry-derived moderate-to-vigorous intensity physical activity and sedentary time for the 54 participants with valid data, and the 31 participants who participated in qualitative interviews. Data are mean ± standard deviation.

Variables	All (n = 54)	Sub-Sample (n = 31)	*p*-Value from T-Test Comparison between All Participants and the Sub-Sample	*p*-Value from T-Test Comparison between Accelerometer and IPAQ
IPAQ MVPA (MET–min/week)	675.7 ± 532.0	764.5 ± 549.9	*p* = 0.211	*p* < 0.001
IPAQ ST (min/day)	577.8 ± 860.7	723.6 ± 1155.0	*p* = 0.205	*p* < 0.001
Accelerometery MVPA (MET min/week)	1253.0 ±771.2	1409.3 ± 909.9	*p* = 0.195	
Accelerometery ST (min/day)	551.4 ± 95.0	566.7 ± 95.4	*p* = 0.314	

ST: Sedentary Time; MET: Metabolic Equivalent of Task.
